# Protocol for microplastic pollution monitoring in freshwater ecosystems: Towards a high-throughput sample processing - MICROPLASTREAM

**DOI:** 10.1016/j.mex.2021.101396

**Published:** 2021-05-25

**Authors:** Aline Reis de Carvalho, Camille Van-Craynest, Louna Riem-Galliano, Alexandra ter Halle, Julien Cucherousset

**Affiliations:** a CNRS, Université Toulouse III - Paul Sabatier, UMR 5623 Laboratoire des Interactions Moléculaires et Réactivité Chimique et Photochimique (IMRCP), 118 route de Narbonne, Toulouse 31062, France; b CNRS, Université Toulouse III - Paul Sabatier, IRD, UMR 5174 Laboratoire Évolution et Diversité Biologique (EDB), 118 route de Narbonne, Toulouse 31062, France

**Keywords:** Organic matter, Digestion, Polymers, FTIR

## Abstract

Robust and reproducible quantification of microplastic pollution in freshwater ecosystems requires the processing of a large amount of samples collected in varying environmental conditions. Such samples are characterized by a high amount of organic matter compared to microplastics and are highly variable in terms of the quantity and the composition of matrices, requiring a standardized analytical protocol for sample treatment and analysis. However, two important and time-consuming steps for microplastic recovery are the elimination of organic matter and microscopic inspection of samples. Here, we developed and validated a protocol, targeting particles with length ranging from 700 µm to 5 mm, that includes a double-step digestion of organic matter, consisting of incubation with potassium hydroxide followed by hydrogen peroxide solutions, and two stereomicroscopic analyses. In addition, we developed several technical improvements allowing reducing the time needed to process samples, such as the design of an adapted filter-cap to improve the content transfer. The absence of physical and chemical alterations in the investigated microplastic pellets and the average reduction of 65.8% (± 9.59 SD) of organic matter in real samples demonstrated that our protocol is fit for purpose. We recommend a second stereomicroscopic analysis to avoid underestimating microplastic concentration and particle size distribution biased towards larger particles. When used for a large-scale monitoring of microplastic pollution, this protocol resulted in an estimated time of 38 h for one person for the treatment of a batch of 24 samples, allowing a higher throughput sample processing and reproducible quantification.

• *Protocol customization towards high-throughput sample processing*

• *Double step digestion to improve organic matter elimination*

• *Importance of stereomicroscopic analysis for microplastic recovery*

## Graphical abstract

**Figure:**
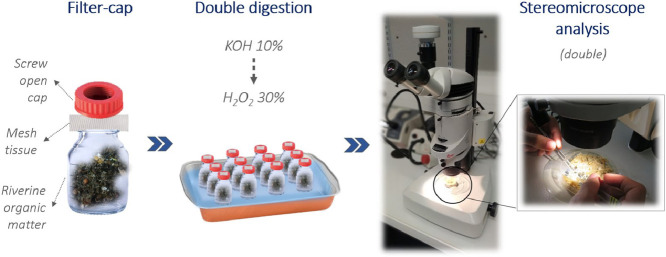

Specifications Table

**Table:** 

Subject Area:	Environmental Science			
More specific subject area:	*Microplastic pollution*			
Method name:	MICROPLASTREAM			
Name and reference of original method:				
Resource availability:	Material	Specifications	Quantity	Observations
	96-well plates	–	–	–
	Aluminum tray	–	–	–
	ATR-FTIR spectroscope	Thermo Nicolet 6700, Thermo Fisher Scientific	–	–
	Balance	AT21 Comparator, *d* = 0.001 mg, Mettler Toledo	–	For particles
	Bottle	250 mL, GL-45	24	–
	Filter paper	–	–	–
	Heating plate	–	–	–
	Hydrogen peroxide	30% (w/w) solution	–	CAS: 7722–84–1
	Nitex tissue	500 µm	0.5 m^2^	–
	Open screw cap	GL-45	24	–
	Petri dish	8 cm diameter	–	Similar quantity as samples
	Potassium hydroxide	> 85% purity, pellets	–	CAS: 1310–58–3
	Sieve	500 µm	1	Stainless
	Stereomicroscope with camera	Leica MZ 75 and Nikon SMZ 800	–	Equipped with a digital camera
	Thermometer	–	–	–
	Tweezers	–	2	Straight and curved ones

**Method details**

## General context

Environmental microplastic pollution, i.e. plastic particles smaller than 5 mm [1], is an emerging concern due to their potential impacts on organism health, biological diversity and ecosystems [6,16]. Microplastic pollution has primarily been quantified and characterized in marine ecosystems, considered as a final sink of these particles [8,26]. Freshwater ecosystems (streams, rivers and lakes) are also extremely important in the dynamic of microplastic pollution because they act as a main source and are responsible for its transport and retention [26]. Accordingly, an increasing number of studies have focused on microplastic pollution in freshwater ecosystems [12,13]. For a robust assessment of microplastic pollution, studies performed at large spatial (e.g. across watersheds) and temporal (e.g. across months and seasons) scales, resulting in high amount of samples, are needed. Therefore, the development of a simplified and reproducible protocol for sample processing is crucial. The detection of microplastics in environmental matrices faces two crucial issues: reduction of matrices effects without altering the target particle, and the unequivocal identification of the targets [29]. However, the quantity and content of freshwater matrices, notably in terms of organic matter and level of microplastic pollution, are highly variable, limiting our ability to settle long-term monitoring of microplastic pollution. The establishment of a standard and high throughput protocol for the quantification and characterization of microplastic in freshwater ecosystems should therefore consider these aspects [17].

## Protocol for sample processing

Current processing of environmental samples for microplastic detection consists of sample collection followed by sample treatment to reduce organic matter content and sample analysis for particles identification [17,22]. The diversity of organic matter composition has led to the development of distinct protocols for sample digestion, either for marine water (e.g. NOAA), sediments or aquatic organisms samples [2,[18], [19], [20]. Importantly, protocol selection or the adaptation of an existing protocols should take into account the purposes of the study and the studied matrix. Organic matter elimination through digestion might be achieved by incubating the sample with an acidic or alkaline solutions, such as potassium hydroxide (KOH), with peroxides solutions, such as hydrogen peroxide (H_2_O_2_) or through an enzymatic reaction. Opposite findings regarding the efficacy of organic matter digestion through different protocols and matrices have already motivated the use of a multiple-step digestion, with different reagents [7,21], although a single reagent is still used in many studies [22].

**Fig. 1:**
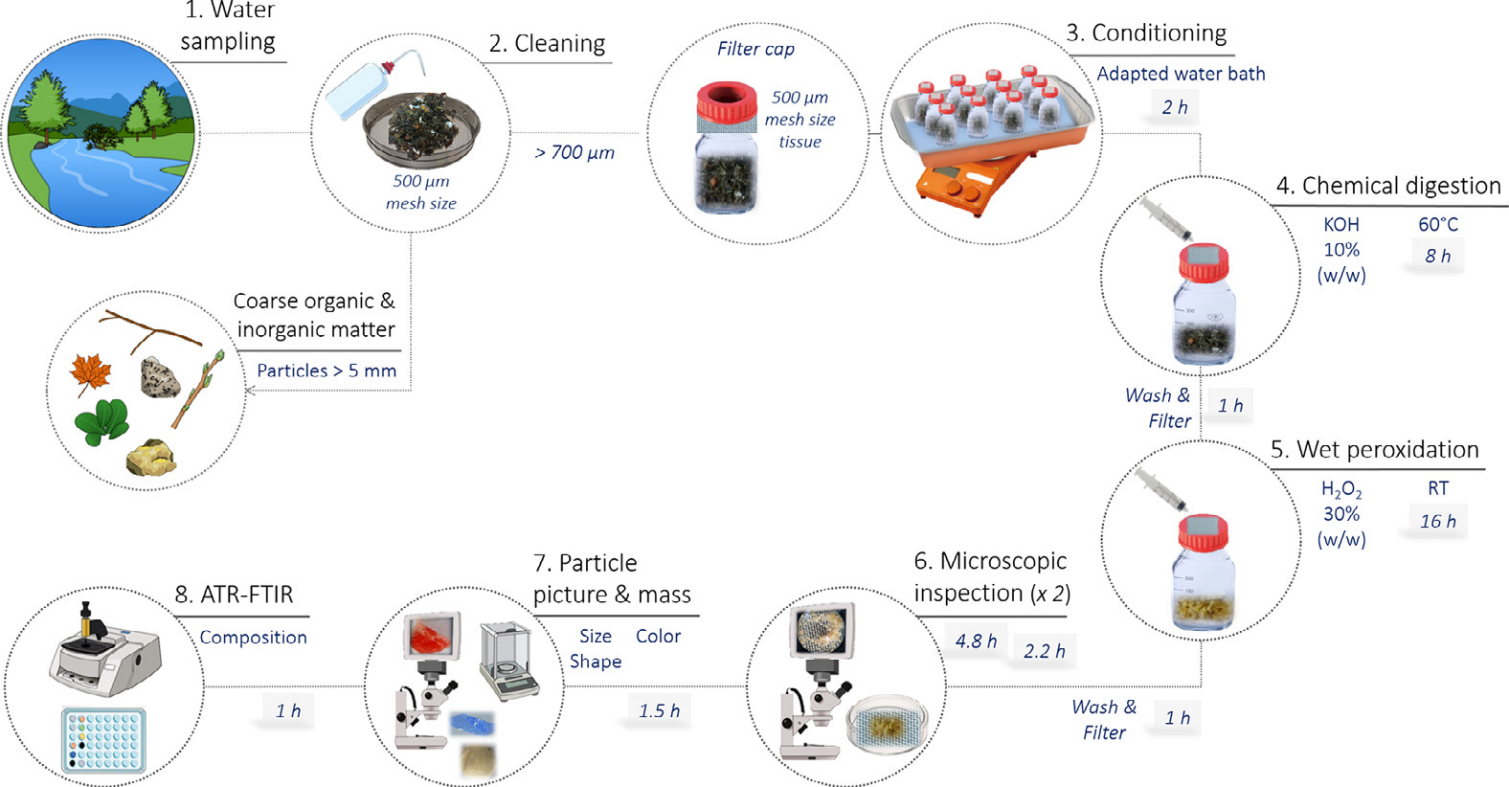
Global overview of the protocol and its different steps. The time displayed represent the analyses of a batch of 24 samples.

In this study, a double-step digestion consisting of two different reagents, potassium hydroxide (KOH) (pellets, Sigma-Aldrich, USA) 10% (w/w) and hydrogen peroxide (H_2_O_2_) 30% (w/w) (Merck KGaA, Germany) solutions was used to optimize the digestion of the rich and diverse organic matter content in freshwater samples (Fig. 1). KOH and H_2_O_2_ are the two main reagents used for digestion purposes in microplastic monitoring studies [25] and were therefore tested in this protocol. Because a multi-step digestion protocol would require the inclusion of washes and filtrations steps, a customized filter-cap was designed to facilitate content drain-out. The glass bottle was covered with a Nitex tissue (500 µm, similar to the water sampling net), and a commercially available screw open-cap (Fig. 3). A syringe was used to facilitate liquid addition through the tissue. Finally, the critical step of microscopic analysis of samples was verified and we concluded that two stereomicroscope analyses, by two different operators, represent a good compromise between analyses time and particles recovery, both in terms of quantity and characteristics of microplastics. In this protocol, microplastic was defined as particle with a major axis larger than 700 µm (i.e. diagonal of the 500 µm mesh net of sampling device) and smaller than 5 mm, and with composition defined as plastic, comprising synthetic polymers, petroleum-based waxes, tire and wear particles and, paint resins [11]. Fibers were not considered here. Considering the instrumental size limitation associated with the detection and quantification of particles by visual inspection using a microscope [9], the selected size range (700 µm – 5 mm) favors an optimal chemical identification by attenuated total-reflectance Fourier-transformed infra-red (ATR-FTIR) spectroscopy, in which a minimum score of 60% of library match was applied.

1. Sample collection (field sampling)
1.1. Field sample with a Manta trawl of 500 µm mesh (Fig. 2a) and collected the in the cod-end (Fig. 2b).

**Fig. 2:**
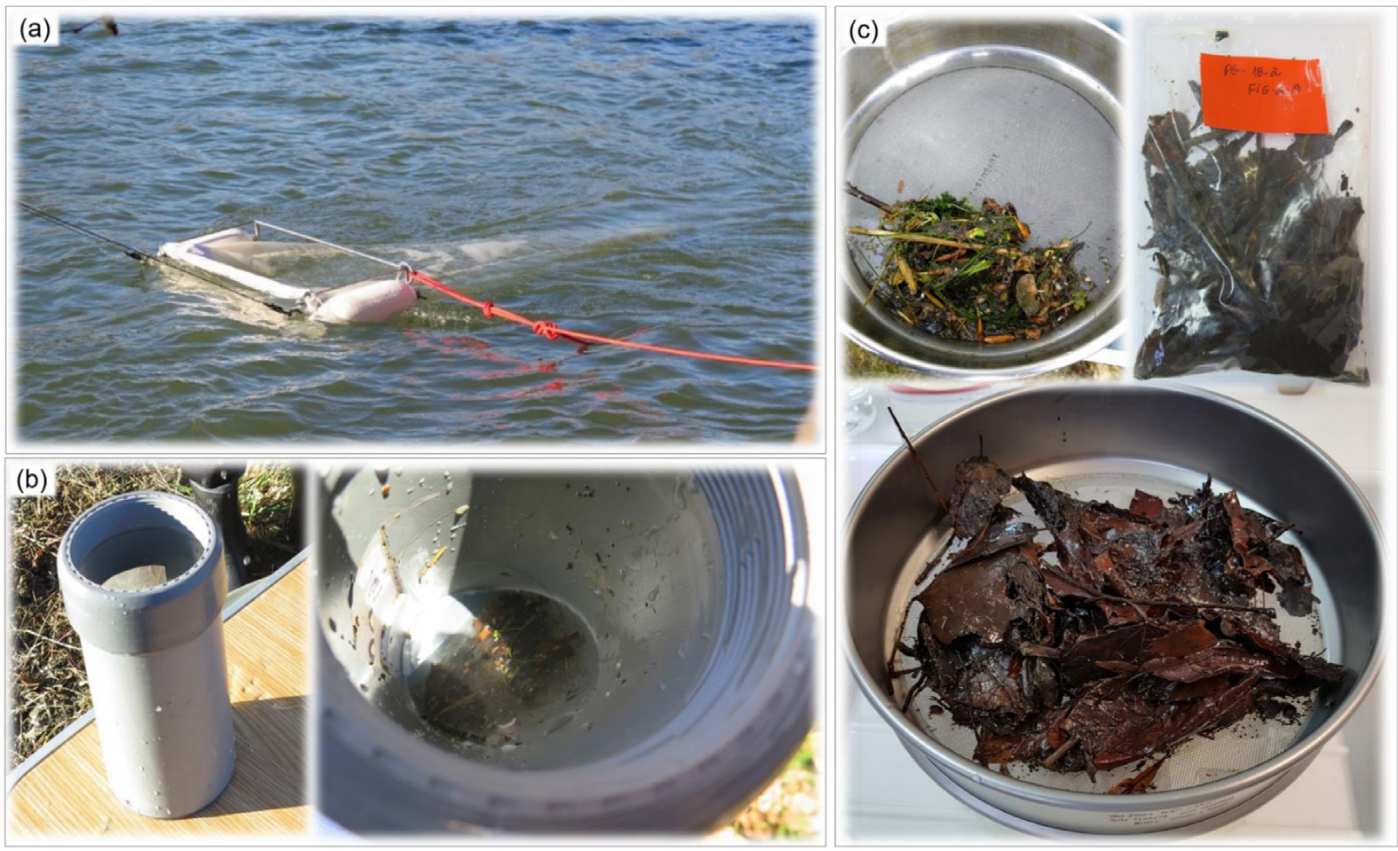
Sample collection in the field with (a) Manta trawl equipped with (b) a removal cod-end. Samples are filtered in the field (c) using a 500 µm sieve and stored in a plastic bag before processing in the laboratory.
1.2. Filter the sample through a 500 µm metal sieve (Fig. 2c).
1.3. Transfer the retained sample into labelled and sealable plastic bags (Fig. 2c).
1.4. Store in the fridge (4 °C) until analyses.
2. Sample treatment (laboratory analysis)
2.1. Measure the wet mass of the sample.
2.2. Over a 500 µm sieve, remove coarse organic and inorganic debris, such as branches, pebbles, leaves and gravels, and particles larger than 5 mm, rinsing with distilled water.
2.3. Transfer the retained content into labelled glass bottles of 250 mL.
2.4. Add KOH 10% (w/w) solution in a proportion of 4 units of volume (mL) for 1 unit of mass of sample (g).

If sample wet mass > 40 g, leading to > 160 mL of reagent, it is recommended to split the sample into several glass bottles to avoid overflow.

**Fig. 3:**
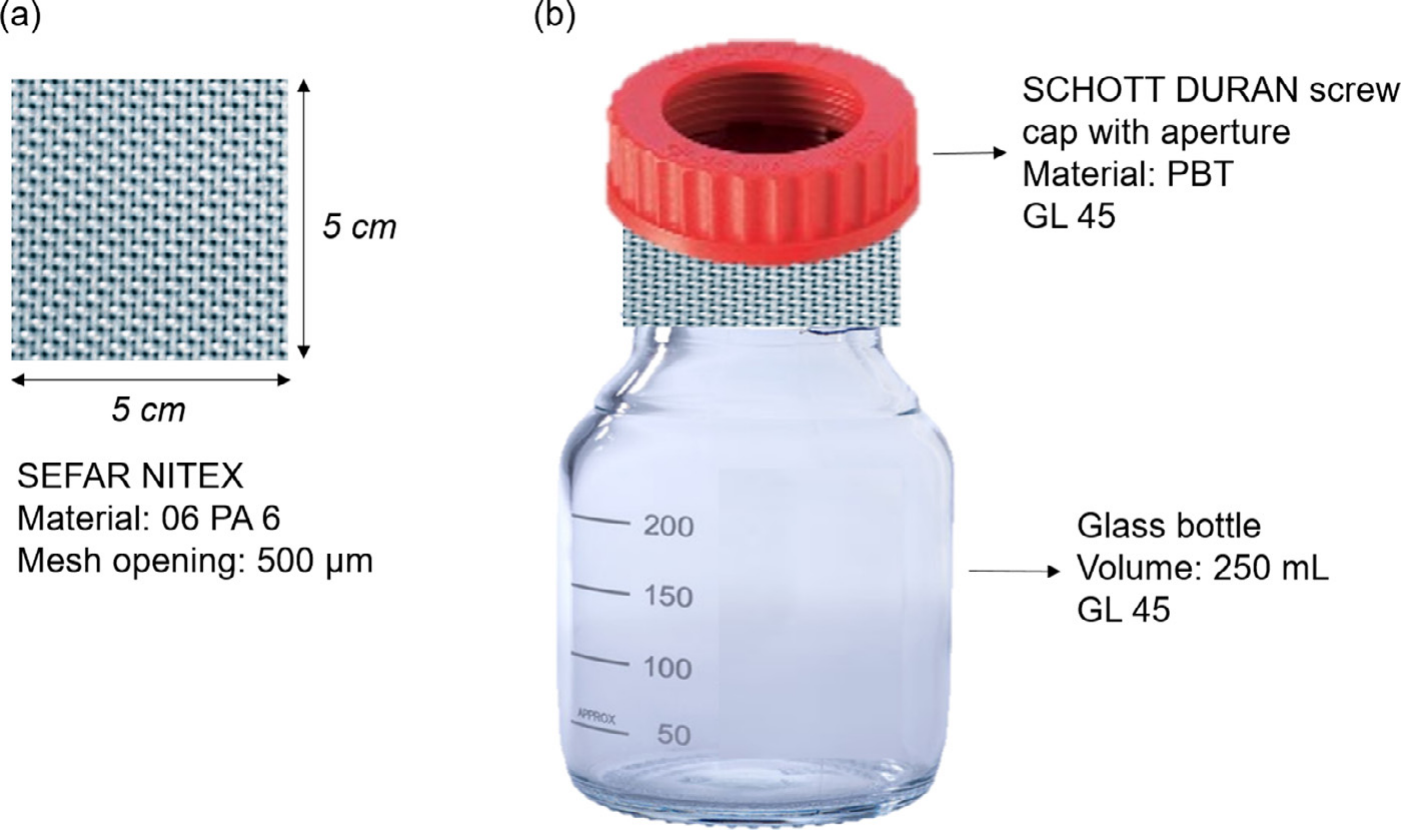
Customized bottles used for sample digestion. (a) A piece of tissue is placed (b) between the screw open and the glass bottle.
2.6. Cover the bottles with aluminum (foil or tray).
2.7. Incubate in a water bath at 60 °C for 8 h.

2.5. Place pre-cut fabrics of Nitex tissue in square format (5 cm x 5 cm) on the top of bottles and use open screw caps to close (Fig. 3).

- Heating at 60 °C was proposed to reduce incubation period [5].

- An adapted aluminum tray bath was employed (Fig. 1, step 3), although an inox tray is recommended due to its higher resistance to oxidation.
- Monitor temperature with a thermometer immersed in a similar glass bottle filled with water only.

- Verify water level in the bath at every 2 h and refill when needed.

**Fig. 4:**
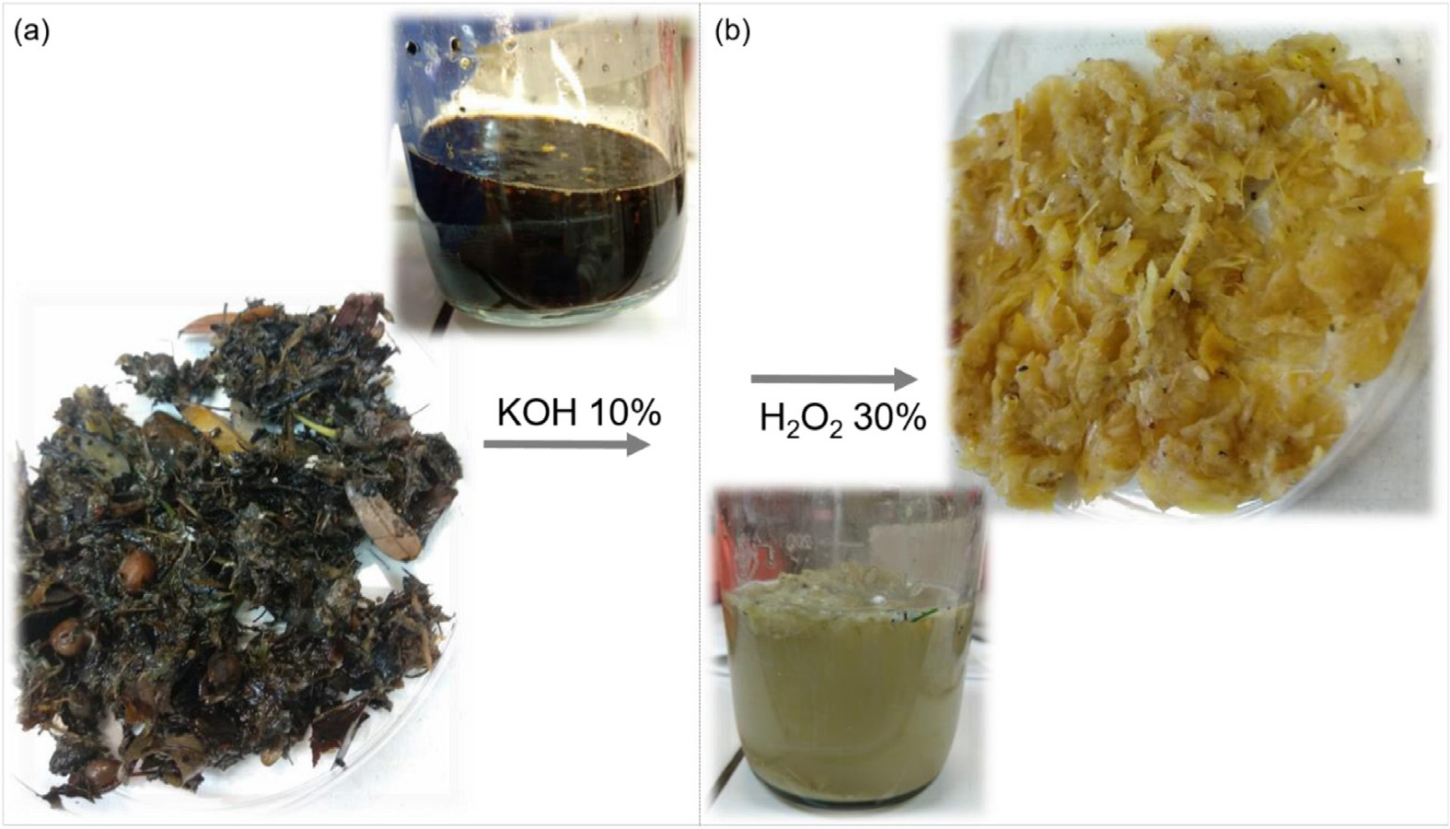
Changes in sample characteristics induced by the double digestion protocol: (a) before and (b) after incubation with potassium hydroxide solution followed by hydrogen peroxide solution.
2.11. Remove the liquid in the bottles by pouring them.
2.12. Repeat steps 2.8 to 2.11 three times minimum or until obtaining a clear rinsing liquid.
2.13. Add H_2_O_2_ 30% (w/w) solution until fully covering the whole sample (Fig. 1, step 5).
- This step should be performed with caution once this process may result in a highly reactive mixture.
2.14. Incubate overnight at room temperature (16 h equivalent).

2.8. Remove the liquid in the bottles by pouring through the tissue.
2.9. Add 40 mL of distilled water with a syringe through the tissue (Fig. 1, step 4).
2.10. Shake and stir the bottle to enhance the washing (Fig. 4a).

- Due to the reactive mixture, samples were not heated. Then, the incubation period was slightly longer.

2.15. Repeat steps 2.8 to 2.11 (Fig. 4b).
2.16. Remove the filter-cap, place it upside-down and filter the sample through the tissue, adding water to remove all remaining content in the bottle (Fig. 5a).

**Fig. 5:**
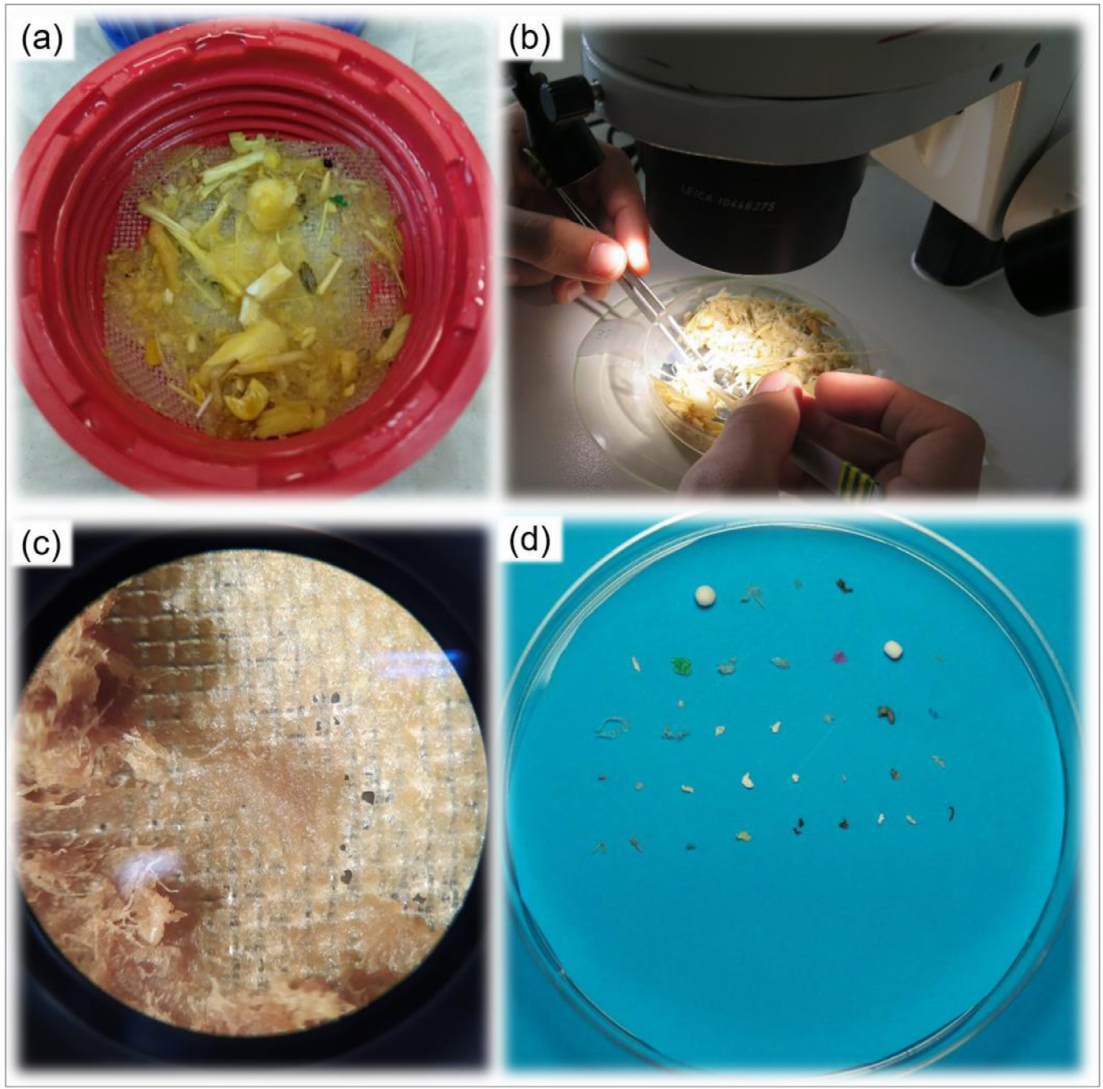
Sample processing after organic matter digestion: (a) transfer to the open cap, (b) microscopic inspection and (c) magnified view and (d) recovered particles.
2.17. Place the tissue with the retained sample in labelled Petri-dish (8 cm diameter) and store at room temperature.
3. Sample analysis (laboratory analysis)
3.1. Analyze the petri-dish under a stereomicroscope (14-fold magnification suggested) (Fig. 1, step 6, Fig. 5b and 5c) and select potential plastic particles, placing them temporarily in a new identified petri-dish (Fig. 5b).

- The time of analysis may strongly vary depending on the amount of remaining organic matter and microplastic concentration.

3.2. Repeat step 3.1.

- To reduce the risk of missing microplastic through manual selection and to avoid potential bias in detection (e.g. particle color, visual appearance), we recommend a second stereomicroscope analysis by a different operator.

- We also recommend to randomize the order of processed samples.

3.3. Picture each particle together with a ruler or size reference and store them individually in a pre-identified petri-dish (Fig. 1, step 7).

- A 96 well-plate is recommended for storing, at room temperature, individually all particles until further analyses.

**Fig. 6:**
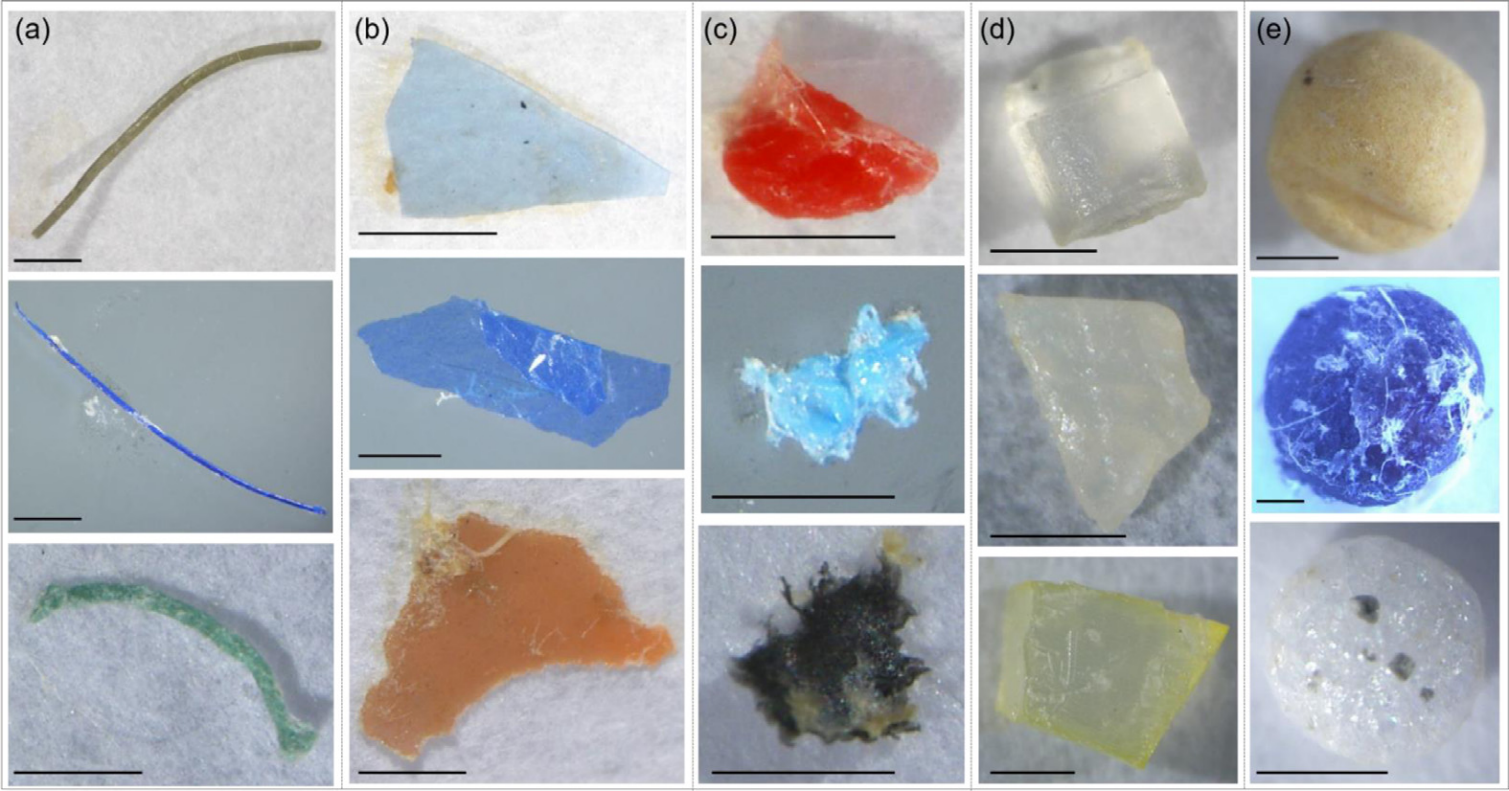
Illustrations of the five categories of particle shape used: (a) line, (b) film, (c) fragment, (d) pellet and (e) sphere. The black line represents 1 mm.

3.4. Categorize the shape of each particle into one of five predefined categories (Fig. 6) adapted from Zobkov [31]:

(a) line: thin elongated items with one dimension significantly greater than the other two;
(b) film: sheets, with their thickness significantly lower than other two dimensions;
(c) fragment: pieces of thick plastics of irregular shape with all three dimensions comparable;
(d) pellet: pieces of regular and non-rounded shape or primary produced particles;
(e) sphere: three dimensional items of spherical shape.

3.5. Using a picture software such as ImageJ [24], measure the two main orthogonal axes in the picture of each particle (i.e. maximal length and height).

- The particle width may be estimated considering each particle shape category [15] .

3.6. Measure the mass (nearest 0.001 mg) of each particle individually and store them back in the same location within the well plate (Fig. 1, step 7).
3.7. Analyze each particle by ATR-FTIR spectroscopy (Fig. 1, step 8).

- Compare the spectra found for each particle with a spectrum library (open source program available, [3]) to assign a composition to each particle.

## Customization and verification essay

**Fig. 7:**
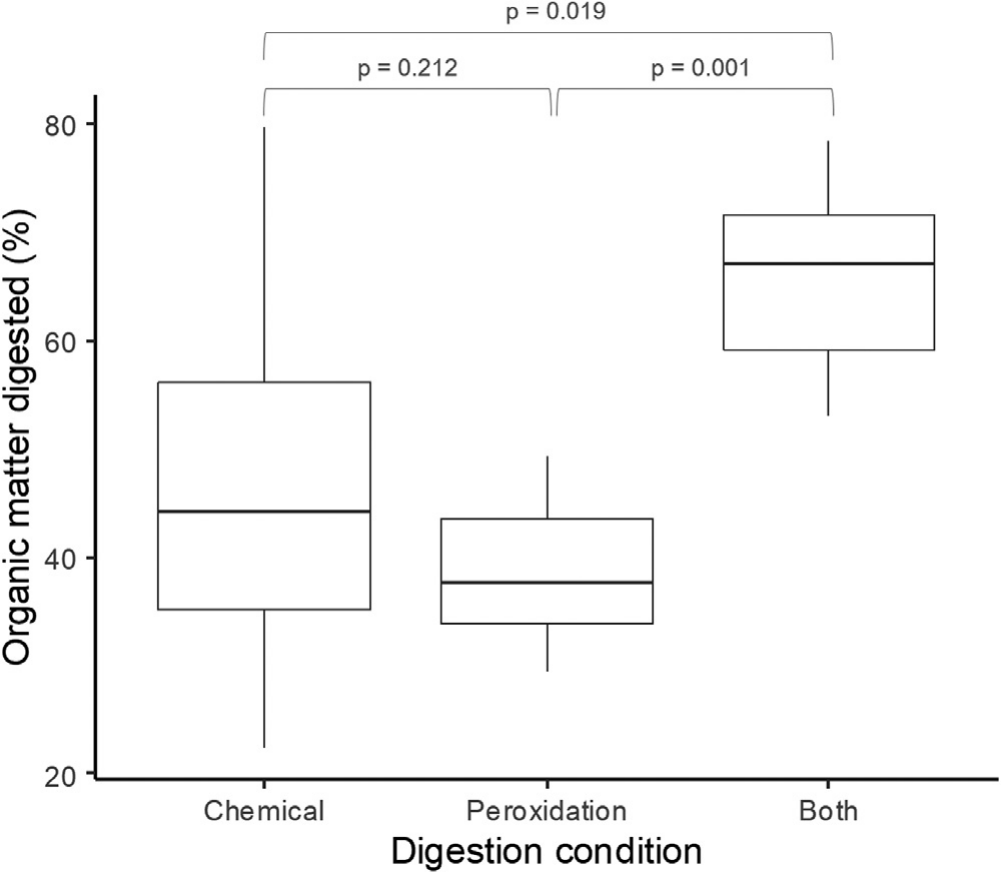
Organic matter digested (%) by the digestion protocols.

In this study, the efficacy of organic matter digestion was quantified using samples collected from the same catchment (*n* = 35) and randomly submitted to three different digestion protocols: two single-reagent digestion (single step; chemical digestion: KOH 10% 60 °C, 24 h and wet peroxidation: H_2_O_2_ 30% room temperature - RT, 24 h) and one double-step digestion (KOH 10% 60 °C followed by H_2_O_2_ 30% RT, totalizing 24 h). We measured sample wet mass before and after digestions and calculated digestion efficiency as the percentage of wet mass loss. We found that the double digestion protocol (*n* = 6) allowed the elimination of, on average, 65.8% (± 9.59 SD) of mass, significantly more efficient than the single ones, with 43.5% (± 15.2 SD) digested for KOH (*n* = 19) and 39.4% (± 7.29 SD) for H_2_O_2_ protocol (*n* = 9) (Kruskal test, χ^2^ = 10.845, *p* = 0.004). No difference was found between the two single protocols (post-hoc comparison, *p* = 0.212) (Fig. 7). The reduction of the organic matter content together with the bleaching effect caused by the wet peroxidation step greatly facilitate the subsequent visual inspection of samples (Fig. 4).

**Fig. 8:**
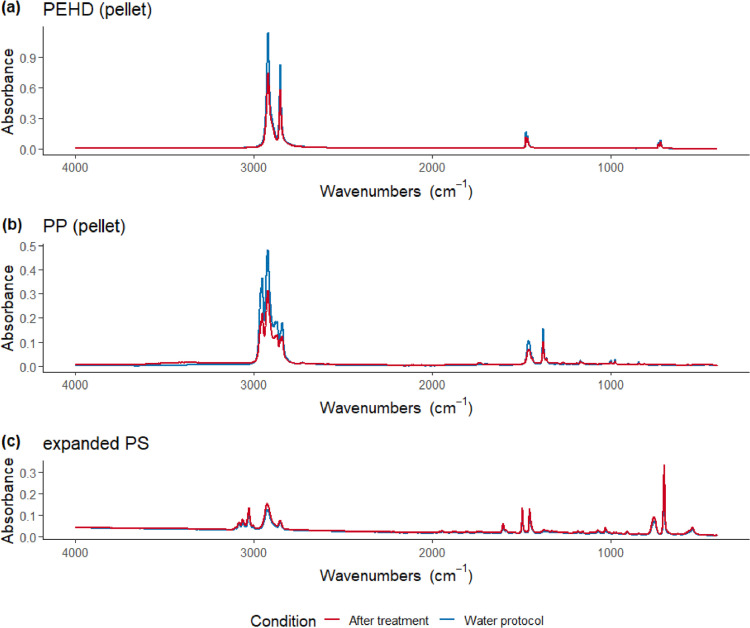
Examples of ATR-FTIR spectra in control condition (blue line) and after digestion protocol (red line) for (a) PE high density (HD, *d* = 0.952 g/mL), (b) expanded PS, (c) PP.

**Fig. 9:**
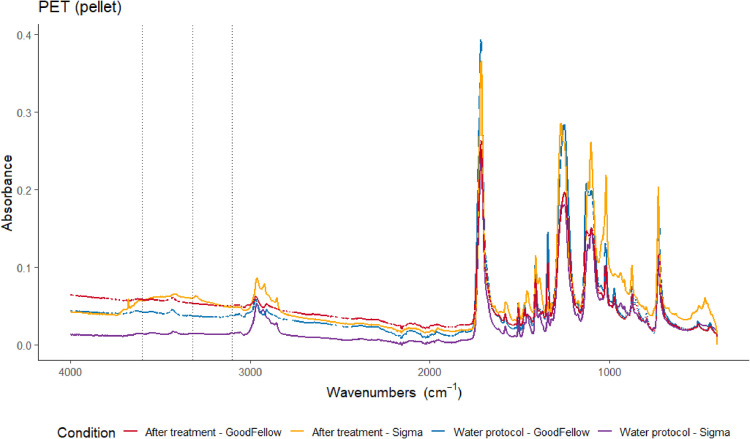
Examples of ATR-FTIR spectra in control condition (blue and purple lines) and after digestion protocol (red and orange lines) for PET from GoodFellow and Sigma manufacturers. Dotted lines in PET spectra (d) represent wavenumbers 3600, 3320 and 3100 cm^−1^.

Although it has been reported that virgin microplastic pellets are not affected by these single protocols [5,14], we quantified potential physical damages through mass changes and chemical modification (assessed by ATR-FTIR) arising from each step of the double digestion protocol. Three to five virgin pellets (1–5 mm) from 12 different synthetic polymers were tested in triplicates: polyethylene (PE) with three different densities, polystyrene (PS), expanded polystyrene, polypropylene (PP), polyethylene terephthalate (PET – from two different manufactures, Sigma and GoodFellow), polyamide 6 and 12, ethylene vinyl acetate, polycarbonate and polyetherimide (**Supplementary Table S1**). The polymers tested represented the main microplastic composition found in environmental samples [28]. No significant alteration that could lead to misidentification was observed in the infrared spectra of particles submitted to digestion protocol when compared with two control conditions, the virgin particle and the treatment with distilled water (Fig. 8). Despite the FTIR spectra of PET after digestion protocol showed a distinct peak at wavenumber 3320 cm^−1^ (Fig. 9, only for pellets from Sigma Aldrich), indicating carboxylic acid and alcohol functional groups (R-OH stretching, 3000–3500 cm ^−1^) [27], all particles were unequivocally identified (Fig. 8) [4]. Similarly, no significant mass changes occurred (Kruskal test, χ^2^ = 1.495, *p* = 0.474), excepted for the two PET batches from Sigma Aldrich (**Supplementary Table S1**), where a significant mass loss of 17.0% (± 5.18 SD) was observed (Kruskal test, χ^2^ = 15.699, *p* = 0.003). Tests with PET pellet from a different manufacture - GoodFellow (**Supplementary Table S1**) showed no significant mass variation following the treatment (98.2% ± 1.81 SD). We highlight that the diversity among plastic formulation might interfere in their chemical stability and further studies regarding potential impacts of this treatment on smaller and/or chemically-altered microplastics are needed.

Microscopic analysis of samples is a critical step for particles detection and we tested the gains obtained with a second and third inspections by different operators. We found that, on average, 23 min (± 10.4 SD) were needed for the first inspection of a sample and that it allowed to recover 91.1% of particles found in the sample. The second and third inspections lasted 5.6 min (± 1.9 SD) and 6.4 min (± 2.0 SD), respectively, and allowed to recover 6.7% and 2.3% of detected particles, respectively

## Applying the protocol to microplastic pollution monitoring

**Fig. 10:**
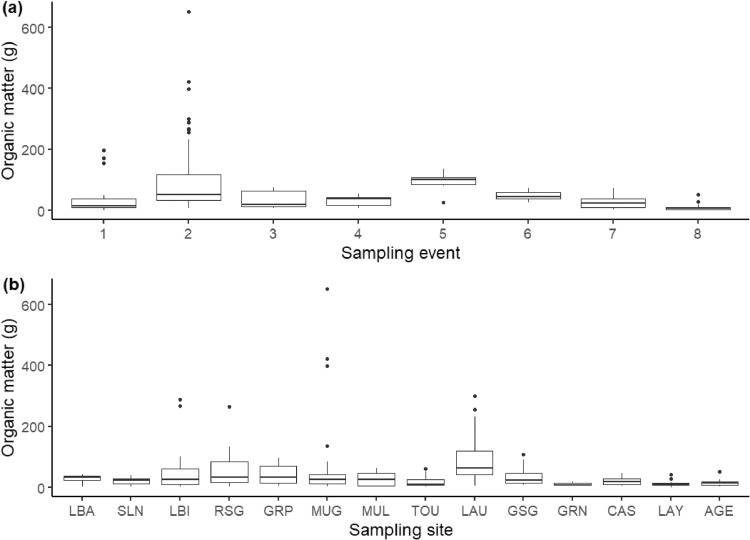
Organic matter mass (g) collected in the samples across (a) sampling events and (b) sampling sites.

The protocol was applied to a total of 204 samples collected in fourteen sites, in triplicates, in the Garonne catchment from February to October 2019. Important temporal (Fig. 10a) and spatial (Fig. 10b) variations of organic matter were observed, both in terms of quantity and composition. On average, sample wet mass was 45.1 *g* ± 76.4 SD. Samples containing a large amount of organic matter were divided (see step 2.4) to obtain a similar mass, resulting in a total of 290 samples in the end. Batches of 24 samples were processed, and the entire processing of a given batch lasted, on average, 38 h (Fig. 1). We found that the digestion protocol finally removed 56.3% ± 25.8 SD of organic matter.

**Fig. 11:**
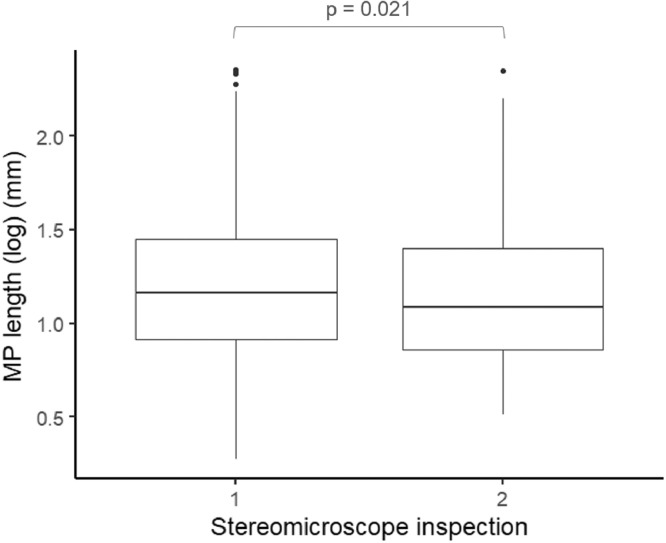
Length (log-transformed) of microplastics recovered during the first and the second stereomicroscope inspections.

The first stereomicroscopic inspection lasted, on average, 13.2 min (± 7.91 SD) and recovered 87% of particles. The second inspection, by a different operator, lasted 5.71 min (± 2.79 SD), representing 5.8% of the total time spent with one sample and 13% of the recovered particles. There was no significant difference in particle color and shape between the two inspections. However, a significant difference was observed regarding particle composition, i.e. plastic or not plastic (χ^2^-test, χ^2^ = 4.091, *p* = 0.043), with higher percentage of non-plastic recovered in the second inspection (14.67% against 19.99%). No difference was found regarding microplastic composition (Fisher test, *p* = 0.894). Independently of particle composition, particles recovered during the second inspection were significantly smaller than those recovered during the first inspection (lmm, χ^2^ = 5.288, *p* = 0.021) (Fig. 11).

Based on these results, we recommend a double-step digestion and a double stereomicroscope inspection by a different operator in order to facilitate sample inspection and avoid bias in concentration and characteristics while quantifying microplastic pollution. This protocol was optimized for our objectives and the environmental matrix found in river surface water. Further adaptations comprising other matrices and/or microplastic smaller than 700 µm are in perspective. In the case of smaller microplastics and because of the instrumental size limitation of ATR-FTIR, other analytical techniques might be applied to guarantee the unequivocal identification of particle composition, such as micro-FTIR (FTIR combined with an optical microscope), Raman or thermoanalytical methods, e.g. pyrolysis coupled to gas chromatography and mass spectrometry (pyr-GC–MS) [9,30]. In the case of smaller microplastics, it is also important to be careful with mesh size in sampling devices as they can lead to net clogging and underestimation of microplastic pollution [4]. Finally, to ensure the robustness of future microplastic pollution monitoring, we also identify a need to improve our knowledge related to the initial step of the process, i.e. field collection and to fully understand the role of small spatial (i.e. lateral and vertical variability) and temporal (e.g. diurnal changes) variations on our estimate of microplastic pollution.

## Statistical analysis

In the verification essay, we used Kruskal-Wallis test to verify if the digestion of organic matter (percentage) differed between digestion protocols and pairwise comparisons were performed with Wilcoxon test. In the microplastic resistance essay, we used the same test to verify differences in microplastic mass due to digestion protocols. To compare the composition of particles, i.e. plastic or not, among the two stereomicroscope inspections, χ2 tests were performed. Fisher Exact tests were applied to compare particle color (seven categories), particle shape (five categories) and composition (eleven categories) among the two stereomicroscope inspections due to limited amount of particles in some categories. The relationship between particle size (log-transformed) with stereomicroscope inspections were tested using a linear mixed-effect model (lmm) with particle color and polymer type as random factor. All statistical analyses were performed using R v.4.0.2 (R [23]). Significant levels of mixed effects model were obtained using the ‘Anova’ function in the car package [10]. Assumptions of linearity and homogeneity of variances on residuals from all models were checked visually.

## Declaration of Competing Interest

The authors declare that they have no known competing financial interests or personal relationships that could have appeared to influence the work reported in this paper.

## Appendix Supplementary materials

Image, application 1
