# Supplementary material for: Protocol for microplastic pollution monitoring in freshwater ecosystems: Towards a high-throughput sample processing - MICROPLASTREAM
Source: MethodsX. 2021 May 25;8:101396. doi: 10.1016/j.mex.2021.101396 (PMC8374496; doi:10.1016/j.mex.2021.101396)
Supplement: Supplementary file 1 [file mmc1.docx]

**Supplementary material *and/or* Additional information:**

Table S1. Polymers references for resistance test

|  | Polymers | CAS | Source | Code | Lot # | OBS |
| --- | --- | --- | --- | --- | --- | --- |
| 1 | Polypropylene | 9003-07-0 | GoodFellow | LS464294 | PP306320/4 |  |
| 2 | Polyethylene low density 1 | 9002-88-4 | Aldrich | 428078 | 07730MEV | d=0.918 g/mL |
| 3 | Polyethylene low density 2 |  | Aldrich | 428043 | MKBL3627V | d=0.925 g/mL |
| 4 | Polyethylene high density |  | Aldrich | 427985 | MKBQ2137V | d=0.952 g/mL |
| 5 | Polystyrene | 9003-53-6 | GoodFellow | LS464294 | ST316310:4 | - |
| 6 | Expanded polystyrene (ePS) | - | *packaging* | - | - | - |
| 7 | Polyethylene terephthalate | 25038-59-9 | Sigma | 429254 | MKBV4092V | - |
|  |  |  | Sigma | 429254 | MKCF1408 | - |
|  |  |  | GoodFellow | LS511753 IM | ES306312/1 | - |
| 8 | Ethylene vinyl acetate | 24937-78-8 | GoodFellow | LS508242 | ET346300/1 | colour: yellow |
| 9 | Polycarbonate | 25037-45-0 | GoodFellow | LS467893 | CT306310/3 | - |
| 10 | Polyamide 6/6 | 32131-17-2 | Sigma | 429171 | MKBX2257V | - |
| 11 | Polyamide 12 | 24937-16-4 | Sigma | 181161 | MKBQ2716V | - |
| 12 | Polyetherimide | 9002-98-6 | GoodFellow | LS403713 | EI316300/1 | colour: natural |
